# Common and recurrent dysregulated molecular network of placental hypoxia and associated vasculogenesis and angiogenesis in fetal growth restriction

**DOI:** 10.3389/fendo.2026.1729898

**Published:** 2026-02-25

**Authors:** Wei Li, Xiaoyi Bai, Oi Ka Chan, Maran Bo Wah Leung, So Ling Lau, Chi Chiu Wang, Tak Yeung Leung

**Affiliations:** 1 Department of Obstetrics and Gynaecology, Faculty of Medicine, The Chinese University of Hong Kong, Hong Kong, Hong Kong SAR, China; 2 Department of Laboratory Medicine, Maternity and Child Healthcare Hospital of Nanshan District, Shenzhen, China; 3 Reproduction and Development, Li Ka Shing Institute of Health Sciences, The Chinese University of Hong Kong, Hong Kong, Hong Kong SAR, China; 4 School of Biomedical Sciences, The Chinese University of Hong Kong, Hong Kong, Hong Kong SAR, China; 5 Chinese University of Hong Kong-Sichuan University Joint Laboratory in Reproductive Medicine, The Chinese University of Hong Kong, Hong Kong, Hong Kong SAR, China; 6 The Chinese University of Hong Kong-Baylor College of Medicine Joint Centre for Medical Genetics, The Chinese University of Hong Kong, Hong Kong, Hong Kong SAR, China

**Keywords:** angiogenesis, fetal growth restriction, hypoxia, placentae, transcriptome, vasculogenesis

## Abstract

Fetuses with fetal growth restriction (FGR) and selective FGR (sFGR) face elevated health risks both before and after birth. Although the underlying pathomechanisms remain unclear, placental dysfunction is recognized as a major contributing factor. By integrating untargeted transcriptomic data from FGR/sFGR placentae, this study identified 69 differentially expressed mRNAs (DEmRs) and eight differentially expressed miRNAs (DEmiRs). Functional enrichment analysis demonstrated significant enrichment in the angiogenesis and vasculogenesis pathways, with the hypoxia-related genes *HIF1A* and *VEGFA* serving as key nodes in the molecular network. Further validation through RNA sequencing (RNAseq), quantitative real-time PCR (RT-qPCR), and immunohistochemistry demonstrated that the expression of the transcriptional regulator *HIF1A* and the angiogenic factor *VEGFA* was upregulated in the placentae of sFGR twins and was significantly associated with clinical severity. Our results indicate that placental hypoxia, vasculogenesis, and angiogenesis via the molecular network of *HIF1A* and *VEGFA* may play an important role in the pathomechanisms of FGR and sFGR.

## 1 Introduction

Fetal growth restriction (FGR) occurs when a fetus does not reach its potential for growth and development *in utero* (1). FGR affects approximately 3%–10% of singleton pregnancies (2, 3). Selective fetal growth restriction (sFGR) occurs specifically in twin pregnancies, where the growth of one twin is restricted while that of its co-twin is normal (4). The incidence of sFGR is estimated at 10%–15% of twin pregnancies (5). Monochorionic (MC) twin pregnancies are more likely to develop sFGR than dichorionic (DC) twin pregnancies due to unequal splitting of an embryo (6). Both FGR and sFGR are associated with increased risks of perinatal morbidity, mortality, and long-term adulthood diseases, such as hypertension and coronary heart diseases (2, 7–15).

The underlying pathomechanism of FGR is still not fully understood. The causes of FGR are diverse and include placental, fetal, and maternal factors (16–18). In singleton pregnancies, the etiology of FGR is highly heterogeneous and arises from complex interactions among placental, maternal, and fetal factors (19). In MC twin pregnancies with sFGR, maternal and fetal factors are shared, and the inter-twin differences are primarily attributable to placental pathological changes and the resulting local functional abnormalities (20). Thus, MC twin sFGR can be regarded as a localized placental disorder arising in the context of shared maternal and fetal factors, in which major confounding variables such as fetal genetic background and maternal systemic factors are effectively controlled. Therefore, sFGR represents an optimal model for investigating the placental contribution to FGR. Studies have attempted to discover any genetic or epigenetic involvement in the pathogenesis of sFGR using transcriptome analysis, but they had several limitations. Firstly, studies on human placentae of twin sFGR pregnancies using whole-genome RNA are sparse, and their sample sizes are small (typically less than 10) (20–24). As a result, the number of differentially expressed genes (DEGs) identified in studies focusing solely on either FGR or sFGR is typically limited, which restricts the power of subsequent functional enrichment analyses. Secondly, the majority of published studies have independently analyzed the placental transcriptome of either singleton FGR or twin sFGR, and few have integrated the two diseases. The aim of our work was to integrate previously published, independently generated, untargeted placental transcriptomic datasets from FGR and sFGR pregnancies into a unified dataset in order to explore the shared and core molecular mechanisms underlying placental dysfunction across these two diseases. Bioinformatics analysis is a useful tool for the integration of RNA sequencing (RNAseq) and microarray data to identify the significant hub genes involved in the pathogenesis of diseases. By using this analysis, differentially expressed hub genes have been identified in the development of other pregnancy-related diseases, such as recurrent miscarriage and preeclampsia (PE) (25, 26).

In this study, we aimed to study significant changes in the gene expression in the placentae of FGR and sFGR in order to understand the underlying molecular pathomechanisms. Here, we firstly searched and identified the potential dysregulated placental genes from the literature. Subsequently, through Gene Ontology (GO), data enrichment, and molecular network analyses, hub genes were selected for validation. Finally, possible pathomechanisms were proposed based on the results. Our study demonstrated that hypoxia-, vasculogenesis-, and angiogenesis-associated pathways via the molecular network of *HIF1A* and *VEGFA* play an important role in the placental pathomechanism of FGR.

## 2 Materials and methods

This study has four parts: 1) Literature search and identification of the potential dysregulated placental genes of FGR and sFGR. 2) Functional annotations such as GO, molecular pathways, and networks were generated by data enrichment and analysis of the identified differentially expressed mRNAs (DEmRs) and targeted genes with differential microRNAs (DEmiRs). 3) Key nodes and hub genes were validated by RNAseq, quantitative PCR, and immunohistochemistry (IHC) staining analyses using our own sFGR cohort. 4) Based on all the above results, a dysregulated molecular network of the placental pathomechanisms in FGR and sFGR was proposed.

### 2.1 Literature search and analysis

Original studies on transcriptome studies of placentae from FGR and sFGR pregnancies were searched from databases including PubMed, Web of Science, and MEDLINE using the keywords “growth restriction,” “growth restricted,” “discordant fetal growth,” and “discordant intrauterine growth” combined with the MeSH terms “DNA,” “gene,” “transcriptome,” “transcript,” “microarray,” and “PCR.” The keywords “placenta” and “placentae” were further used to restrict the source of tissue samples.

The diagnostic parameters for FGR were defined according to the Delphi consensus criteria (1). These parameters differ between singleton and MC twin pregnancies with FGR (**Table 1**).

**Table 1:** Diagnostic parameters for fetal growth restriction (FGR) in singleton and monochorionic twin pregnancies.

Parameter	FGR	MC sFGR
Definition	Any of the following: 1) AC/EFW <3rd centile 2) UtA Doppler velocimetry with absent end-diastolic flow 3) AC/EFW <10th centile plus any one of the following Doppler or growth abnormalities: UtA-PI >95th centile UA-PI >95th centile CPR <5th centile Crossing of growth centiles >2 quartiles	EFW <3rd centile Or at least two of the following: 1) EFW of one fetus <10th centile 2) AC of one fetus <10th centile 3) UtA-PI of the smaller fetus >95th centile 4) EFW discrepancy of ≥25%

GA, gestational age; AC, abdominal circumference; EFW, estimated fetal weight; UA, umbilical artery; UtA, uterine artery; CPR, cerebroplacental ratio; PI, pulsatility index; MC, monochorionic.

We included studies employing non-targeted whole-genome transcriptomic levels of either coding RNA (messenger RNA, mRNA) or non-coding RNA (ncRNA), including microRNA (miRNA) and long non-coding RNA (lncRNA), in human placentae of FGR singleton or sFGR twin pregnancies. Only studies published in the English language were included. The exclusion criteria were: animal studies, cell line studies, triplets and higher-order multiple pregnancies, data mixed with singleton and multiple pregnancies, and other placental complications such as PE, placenta previa, abruption, and gestational diabetes. Review articles or papers that used only targeted RNA detections by RT-PCR, Northern blot, and pyrosequencing were also excluded. RNA profiling without validation or with validation, but with the results inconsistent with the RNA profiling, were also excluded from data analysis.

The following data were extracted from the included studies: publication year, country, authors, the platform used for genome-wide analysis and validation, sample size, gestational age, diagnostic criteria, exclusion criteria, cutoff values for transcript expression change, differentially expressed transcripts, expression direction (up- or downregulated), and molecular pathway. Expression fold change (FC) was calculated using the transcript expression ratios between the FGR/sFGR groups and their corresponding control group.

### 2.2 Data enrichment and network analysis

The names of the transcripts were converted into the official gene symbol using the National Center for Biotechnology Information (NCBI) database. The significant DEmRs and DEmiRs were defined by *p* < 0.05. Target mRNAs of the most significant DEmiRs were also searched from the miRTarBase database and were selected if they fulfilled all three strong-evident experiments (27), reporter assay, Western blot, and qPCR; plus one weak-evident experiment, such as microarray, next-generation sequencing, or pulsed stable isotope labeling by/with amino acids in cell culture, among others.

In order to compare the results between FGR and sFGR and between DEmRs and DEmiRs, three datasets—I) DEmRs from FGR studies, II) DEmRs of pooled FGR and sFGR studies, and III) all DEmRs plus target mRNAs of DEmiRs of pooled FGR and sFGR studies—were used for stepwise functional annotations, including Gene Ontology (GO) and Kyoto Encyclopedia of Genes and Genomes (KEGG) pathway enrichment and network analyses. GO and KEGG enrichment was performed using the Database for Annotation, Visualization and Integrated Discovery (DAVID) bioinformatics tool (version 6.8; https://davidbioinformatics.nih.gov/) (28). The statistically significantly enriched GO terms and KEGG pathways, with a *p*-value <0.05 and false discovery rate (FDR) <0.1, were selected for network analysis and validation.

**Figure 1:**
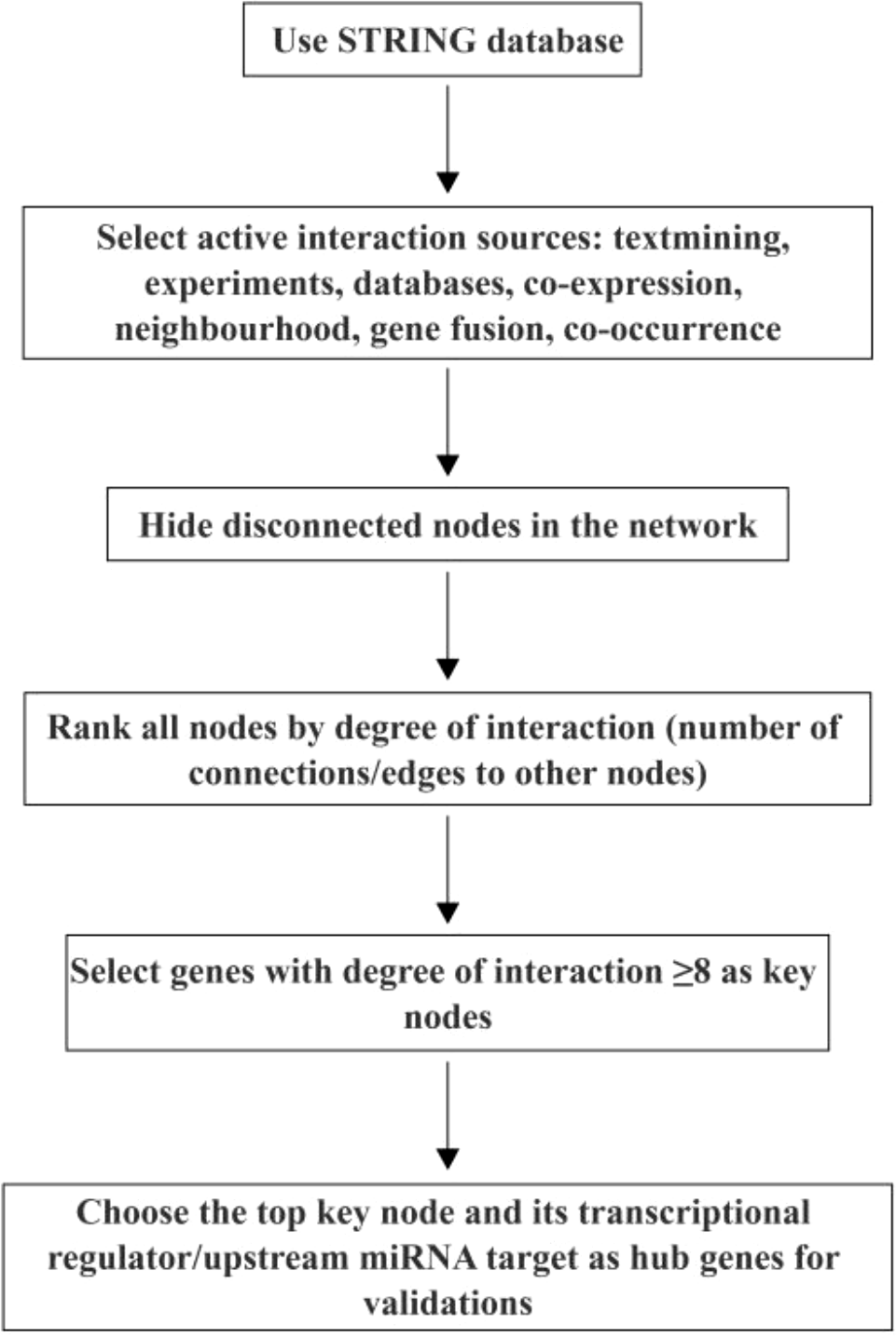
Workflow of hub gene identification via network analysis.

Network analysis was generated by the STRING database (version 11.0, http://string-db.org). Active interaction sources, including text mining, experiments, databases, co-expression, neighborhood, gene fusion, and co-occurrence, were chosen. Disconnected nodes were hidden in the network. All nodes were ranked by degree of interaction, which is the number of connections or edges of the node to other nodes. The genes with a degree of interaction ≥8 were then selected as key nodes. This specific threshold was determined based on the inflection point in the node degree distribution to balance the specificity and sensitivity of the network analysis (29, 30). This effectively identifies biologically relevant hub genes that are significantly enriched in the core FGR pathways and show strong associations with clinical phenotypes (31, 32). The top key node and its transcriptional regulator or upstream miRNA target were chosen as hub genes for validations (**Figure 1**).

### 2.3 Validation studies

Placental tissues collected from our own sFGR cohort were used for validations. Approval of this study was obtained from the Joint Chinese University of Hong Kong–New Territories East Cluster Clinical Research Ethics Committee (CREC reference no. 2014.562) and the Ethics Committee of each participating territory hospital in Hong Kong (NTWC/CREC/1383/14, KC/KE-14-0233/FR-1, and KW/EX-15-006[83-07]). Informed and written consent was obtained from all participants. The investigation also conforms to the principles outlined in the Declaration of Helsinki.

#### 2.3.1 Placental collection

The placenta was examined immediately after delivery. Based on the vascular distribution and amnionic septum, the placental territories corresponding to each twin were identified. Within each twin-specific placental region, a 1-cm^3^ villous tissue sample was excised. The placental tissues were collected from the visually vascular areas rather than from the visually less vascular areas to avoid areas with too many dead cells and calcifications that could affect the assays. After careful separation of the chorionic villi, the tissues were immediately immersed in RNAlater stabilization solution or 10% neutral buffered formalin for subsequent experiments.

#### 2.3.2 RNA sequencing in sFGR placentae

Key nodes were validated using our own dataset of placental transcriptomic profile between five pairs of sFGR twins and two pairs of normal growing controls in MC twin pregnancies by RNAseq. The subject recruitment, placenta collection, and RNAseq and data analyses were as described previously (22).

#### 2.3.3 RT-qPCR in sFGR placentae

The differential mRNA expression levels of the hub genes in the placentae of 18 pairs of MC twins with sFGR pregnancies and seven control pairs of normal twin pregnancies were confirmed by quantitative real-time PCR (RT-qPCR). RNA was extracted from the placentae using the miRNeasy Mini Kit (Qiagen, Valencia, CA, USA) following the manufacturer’s instructions. Complementary DNA synthesis was performed using the High-Capacity cDNA Reverse Transcription Kit (Applied Biosystems-Thermo Fisher Scientific, Waltham, MA, USA), together with SuperScript™ III reverse transcriptase, random hexamers, and RNaseOUT™ Recombinant Ribonuclease Inhibitor (Invitrogen, Carlsbad, CA, USA). RT-qPCR was performed on a fluorescence detector, Applied Bio-systems™ 7900HT (Applied Biosystems-Thermo Fisher Scientific, Waltham, MA, USA), using TaqMan Probe Kit (Applied Biosystems-Thermo Fisher Scientific, Waltham, MA, USA). Gene expression was normalized using the housekeeping gene *18S rRNA*. The primers used were Hs00900055_m1 (for *VEGFA*), Hs00153153_m1 (for hypoxia-inducible factor 1-alpha, *HIF1A*), and Hs99999901_s1 (for *18S*) from Applied Biosystems (Thermo Fisher Scientific, Waltham, MA, USA).

As described previously (22), 2^−Δ^*^C^*^t^ was used to indicate the relative mRNA expression levels of the targeted genes. For each set of twins, the mRNA expression ratio (smaller twin/larger co-twin) = (2^−Δ^*^C^*^t^ of the smaller twin)/(2^−Δ^*^C^*^t^ of the larger co-twin). The mRNA expression ratio was compared between the sFGR group and the control group. To evaluate the association between disease severity and molecular expression, the sFGR cases in our cohort were stratified into two subgroups: complicated sFGR and uncomplicated sFGR. Complicated sFGR was defined as sFGR with pathological cardiotocogram or fetal blood flow, such as the reverse wave of ductus venosus or reverse end-diastolic flow of the umbilical artery. Uncomplicated sFGR was defined as cases meeting the diagnostic criteria for sFGR, but without evidence of overt fetal hemodynamic decompensation or hypoxia-related adaptation. This classification was based on the objective presence or absence of fetal hypoxia or hemodynamic decompensation.

#### 2.3.4 Immunohistochemical staining and *H* score analysis in sFGR placentae

The localization of the protein and the expression levels of the hub genes in the placentae of sFGR and control twin pairs were determined by IHC staining. The cases used for IHC staining were the same as those used for RT-qPCR. Placental sections (5 μl thick) were deparaffinized through graded alcohol and then rehydrated in distilled water. The sections were heated with sodium citrate buffer (10 mM sodium citrate, 0.05% Tween 20, pH 6.0) for antigen retrieval. The activity of endogenous peroxidase was quenched by 3% (*v*/*v*) hydrogen peroxidase in methanol. After blocking, the sections were incubated overnight at 4°C with primary antibodies, including anti-VEGFA (1:50) and anti-HIF1A (1:50) (catalog no. ab1316 for anti-VEGFA and no. ab8366 for anti-HIF1A; Abcam, Cambridge, MA, USA). The secondary antibody was horseradish peroxidase-conjugated rabbit anti-mouse IgG antibody (1:100; catalog no. sc358914; Santa Cruz, Dallas, TX, USA). Diaminobenzidine tetrachloride (DAB) (Sigma, St. Louis, MO, USA) was used for detection. After counterstaining in hematoxylin, the sections were dehydrated in graded ethanol. All mounted slides were examined under a Leica DM6000B microscope (Leica Microsystems, Bannockburn, IL, USA) at ×200 magnification. The intensity of protein expression in the placental sections was evaluated using the *H* score. In each section, the protein staining intensity (0, 1+, 2+, or 3+) was determined for each cell in 10 randomly selected fields. An *H* score was assigned using the formula: [1 × (% cells 1+) + 2 × (% cells 2+) + 3 × (% cells 3+)]. The FC of the protein expression levels between the smaller twin and the larger co-twin is presented as a ratio (smaller twin/larger co-twin) for comparison between the sFGR and control groups.

### 2.4 Statistical analysis

Chi-square or Fisher’s exact test was used for the comparison of categorical variables. The Mann–Whitney *U* test was used to compare the continuous variables in two groups, while the Kruskal–Wallis test was used among three or more groups with pairwise comparisons using the Bonferroni correction method. A *p* < 0.05 was considered statistically significant. SPSS 22.0 software (IBM, Armonk, NY, USA) and R language (3.2.2) were used for data analysis and visualization.

## 3 Results

### 3.1 Differential expressed transcripts

**Figure 2:**
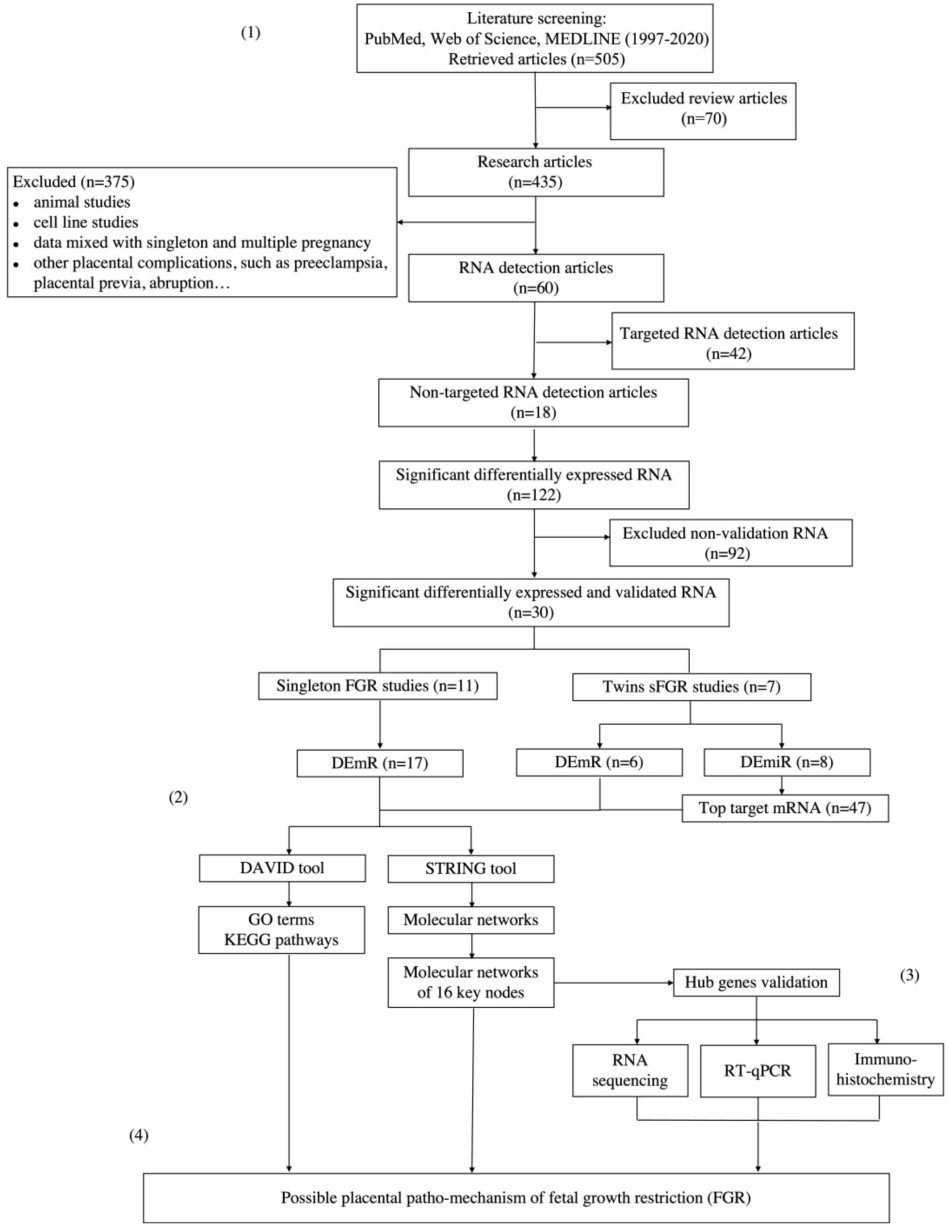
Flowchart of the study. (1) Literature search. (2) Data enrichment and network analysis. (3) Validation. (4) Proposal of possible pathomechanism of fetal growth restriction. *sFGR*, selective fetal growth restriction; *FGR*, fetal growth restriction; *DEmiR*, differentially expressed microRNA; *DEmR*, differentially expressed mRNA; *RT-qPCR*, quantitative real-time PCR.

The workflow of the literature search and selection is shown in **Figure 2**. A total of 18 studies met the criteria and were included in the study (20–24, 33–45), encompassing 11 singleton FGR and seven twin sFGR studies (**Supplementary Table S1**). Among them, 17 DEmRs from the FGR studies and six DEmRs from the sFGR studies were identified (**Supplementary Table S2**). In addition, eight DEmiRs were identified (all from the sFGR studies), of which 47 predicted target mRNAs were identified according to the miRTarBase database (**Supplementary Table S3**).

**Figure 3:**
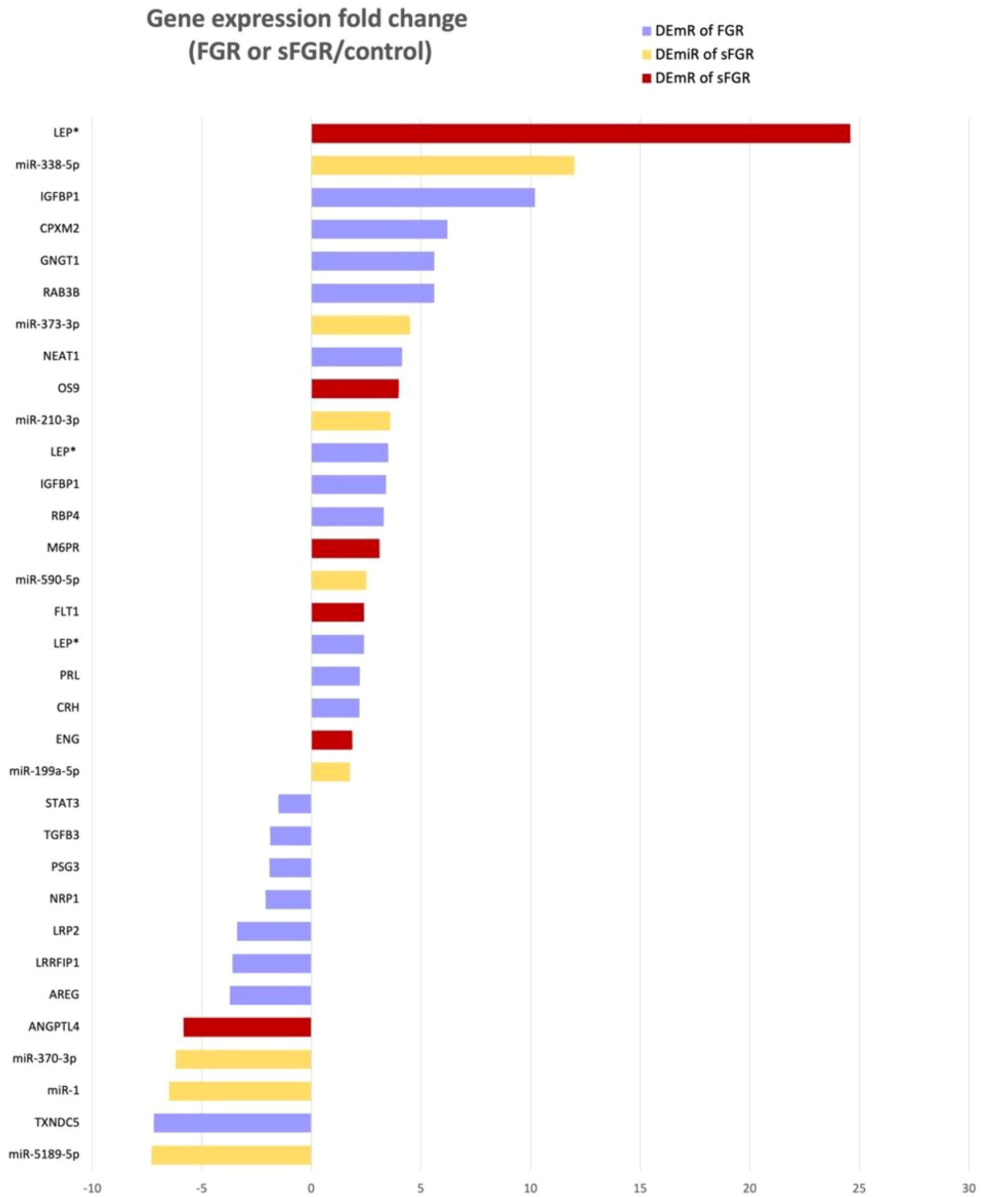
Fold change diagram of the significantly differentially expressed genes in the placentae of fetal growth restriction (FGR) and selective FGR (sFGR). There are 17 significantly differentially expressed mRNAs (DEmRs) from FGR (*blue bar*; two DEmRs overlapped in two studies), six DEmRs from sFGR (*red bar*), and eight significant differentially expressed microRNAs (DEmiRs) from sFGR (*yellow bar*). The fold change is presented as a ratio of the gene expression levels of the FGR or sFGR cases over those of their corresponding controls. *LEP* is marked by an *asterisk*, indicating that it is identified in both FGR and sFGR studies. *LEP*, leptin; *IGFBP1*, insulin-like growth factor-binding protein 1; *CPXM2*, carboxypeptidase X, M14 family member 2; *GNGT1*, G-protein subunit gamma transducin 1; *RAB3B*, Ras-related protein Rab-3B; *NEAT1*, nuclear enriched abundant transcript 1; *OS9*, endoplasmic reticulum lectin; *M6PR*, mannose-6-phosphate receptor; *RBP4*, retinol-binding protein 4; *FST1*, follistatin 1; *PRL*, prolactin; *CRH*, corticotropin-releasing hormone; *ENG*, endoglin; *STATA3*, signal transducer and activator of transcription 3; *TGFB3*, transforming growth factor beta 3; *PSG3*, pregnancy-specific beta-1-glycoprotein 3; *NRP1*, neuropilin-1; *LRP2*, LDL receptor-related protein 2; *LRRFIP1*, LRR binding FLII interacting protein 1; *AREG*, amphiregulin; *ANGPTL4*, angiopoietin-like 4.

The expression FC values of the DEmRs and DEmiRs are shown in a bar chart (**Figure 3**). Among them, 18 RNAs (13 mRNAs and five miRNAs) were upregulated (FC range from 12.0 to 1.75). Leptin (*LEP*) was the most significantly upregulated gene in two FGR studies (FC = 3.5 and 2.4, respectively) and one sFGR study (FC = 24.59). *Insulin-like growth factor-binding protein 1* (*IGFBP1*) was upregulated in two FGR studies (FC = 10.2 and 3.4, respectively). There were 12 RNAs (nine mRNAs and three miRNAs) that were downregulated (FC range from −7.3 to −1.51) in FGR or sFGR when compared with the control.

### 3.2 Vasculogenesis, angiogenesis, and hypoxia enriched transcripts

According to the above findings, three datasets were used for stepwise GO enrichment analysis. This stepwise integration was designed to leverage the complementary pathophysiological profiles of FGR and sFGR and to address the limitation of small sample sizes in individual studies. This approach progressively expanded the gene pool while preserving the core molecular features of FGR/sFGR, thereby enhancing the statistical power for the identification of consistently enriched pathways.

- Dataset I: the 17 DEmRs from FGR studies;
- Dataset II: the 17 DEmRs in dataset I, plus the five DEmRs unique in the sFGR studies (a total of 22); and
- Dataset III: the 22 DEmRs in dataset II plus the 47 target mRNAs of the eight DEmiRs identified in the FGR/sFGR studies (a total of 69).

**Figure 4:**
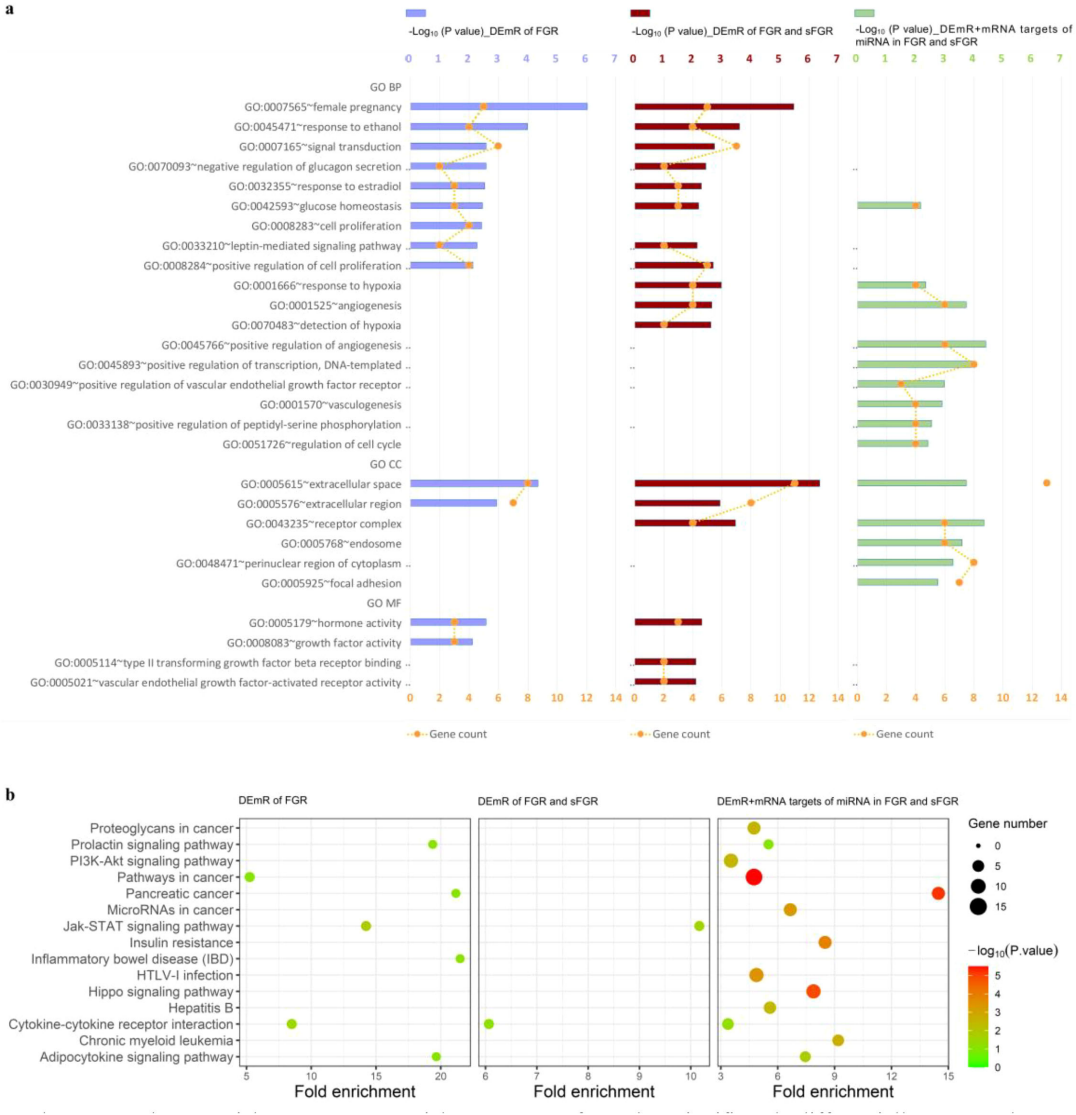
Gene Ontology (GO) and Kyoto Encyclopedia of Genes and Genomes (KEGG) pathway enrichment. **(A)** GO enrichment was performed on the significantly differentially expressed mRNAs (DEmRs) of fetal growth restriction (FGR) (*left*, *medium slat blue*), the DEmRs of FGR and selective fetal growth restriction (sFGR) (*middle*, *red*), and the DEmRs and mRNA targets of the significantly differentially expressed miRNAs of FGR and sFGR (*right*, *green*). Statistically significant GO terms with *p*-value <0.05 and false discovery rate (FDR) <0.1 in the biological process, cellular component, and molecular function branches are displayed. *Bar charts* represent −Log10 (*p*-value) per GO term. *Yellow dots with axes at the bottom* indicate the number of DEmRs identified in each GO term. **(B)** KEGG pathway enrichment was performed for the significantly DEmRs of FGR (*left*), the DEmRs of FGR and sFGR (*middle*), and the DEmRs and mRNA targets of the significantly differentially expressed miRNAs of FGR and sFGR (*right*). *Bubble size* represents the number of DEmRs enriched in the pathway. The *color of the bubble* indicates enrichment significance, which was calculated using −Log10 (*p*-value). *BP*, biological process; *CC*, cellular component; *MF*, molecular function.

The enrichment results are listed in **Supplementary Table S4**. Statistically significant GO terms with *p*-value <0.05 and FDR <0.1, including 18 biological process (BP), six cellular component (CC), and four molecular function (MF) terms, are presented in **Figure 4A**. Among the 18 BP terms, six were related to vasculogenesis, angiogenesis, and hypoxia. The most significant one was positive regulation of angiogenesis (dataset III; *p* = 3.98E−05) and angiogenesis (dataset III; *p* = 1.83E−04), followed by detection of hypoxia, response to hypoxia, vasculogenesis, and positive regulation of vascular endothelial growth factor receptor. Among the six CC terms, extracellular space was the most significantly enriched in all three datasets, which is related to angiogenesis indirectly through the secretory angiogenic regulators vascular endothelial growth factor A (*VEGFA*) and *LEP*. Among the four MF terms, the most significant was hormone activity (dataset III; *p* = 2.67E−03), and the involved DEmRs included *LEP*, prolactin (*PRL*), and corticotropin-releasing hormone (*CRH*). Another term, vascular endothelial growth factor-activated receptor activity, containing the DEmRs neuropilin-1 (*NRP1*) and Fms-like tyrosine kinase 1 (*FLT1*), was also associated with angiogenesis.

The enriched KEGG pathways are listed in **Supplementary Table S5** and presented in **Figure 4B**. Six KEGG pathways were enriched by at least two datasets, including the pathway of cancer, pathway of pancreatic cancer, prolactin signaling pathway, Jak-STAT signaling pathway, cytokine–cytokine receptor interaction, and adipocytokine signaling pathway; however, only the first two pathways were statistically significant, with *p*-value <0.05 and FDR <0.1.

*VEGFA*, a factor stimulating vasculogenesis and angiogenesis, was involved in the two pathways. Moreover, *HIF1A* and other angiogenic and/or vasculogenic regulators, such as Wnt family member 2 (*WNT2*), glycogen synthase kinase 3 beta (*GSK3B*), TGF-beta receptor type-1 (*TGFBR1*), TGF-beta receptor type-2 (*TGFBR2*), transforming growth factor beta 3 (*TGFB3*), cadherin 1 (*CDH1*), and signal transducer and activator of transcription 3 (*STAT3*), were involved in the pathway of cancer. In addition, *TGFBR1*, *TGFBR2*, *TGFB3*, and *STAT3* were also contained in the pathway of pancreatic cancer.

### 3.3 HIF1A and VEGFA as the key node of the molecular network

**Figure 5:**
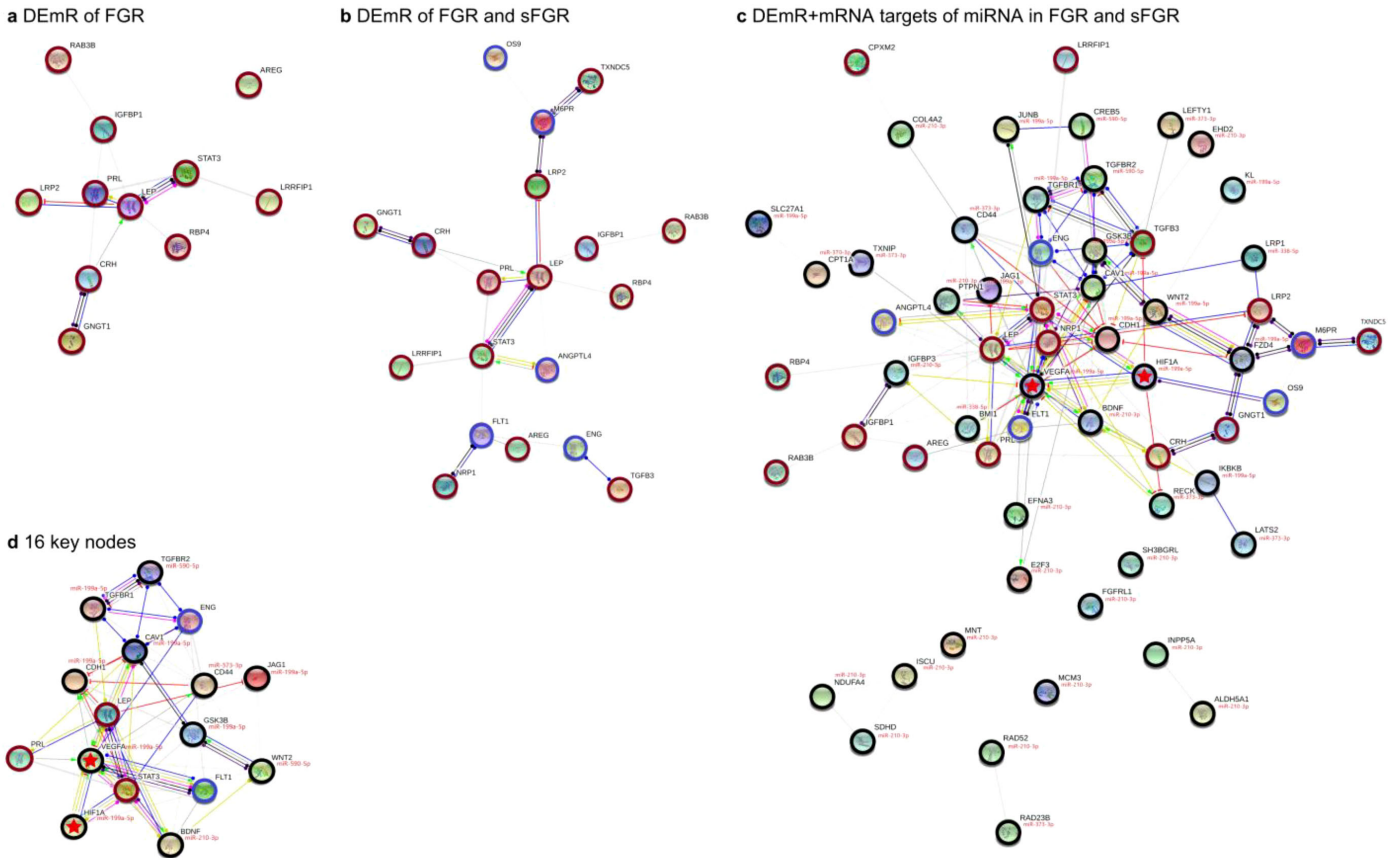
Molecular interaction networks. Molecular interaction network analysis was performed on the significantly differentially expressed mRNAs (DEmRs) of fetal growth restriction (FGR) **(A)**, the DEmRs of FGR and selective fetal growth restriction (sFGR) **(B)**, and the DEmRs and mRNA targets of the significant differentially expressed miRNAs of FGR and sFGR **(C)**, with 16 key nodes **(D)**. Nodes are marked with *different colored circles*: DEmRs of FGR (*red circle*), DEmRs of sFGR (*blue circle*), and mRNA targets of the significantly differentially expressed miRNAs of FGR and sFGR (*black circle*). The miRNA is shown in *red* near its target mRNA. The *color of the edge line* indicates the activity types: activation (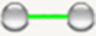), binding (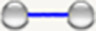), phenotype (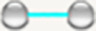), reaction (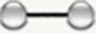), inhibition (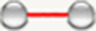), catalysis (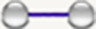), posttranslational modification (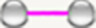), and transcriptional regulation (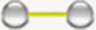). The *shape of the edge line* indicates the activity effects: positive (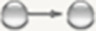), negative (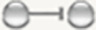), and unspecified (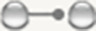). The *edge line width* indicates the relatedness between two nodes. *LEP*, leptin; *IGFBP1*, insulin-like growth factor-binding protein 1; *CPXM2*, carboxypeptidase X, M14 family member 2; *GNGT1*, G-protein subunit gamma transducin 1; *RAB3B*, Ras-related protein Rab-3B; *NEAT1*, nuclear enriched abundant transcript 1; *OS9*, endoplasmic reticulum lectin; *M6PR*, mannose-6-phosphate receptor; *RBP4*, retinol binding protein 4; *FST1*, follistatin 1; *PRL*, prolactin; *CRH*, corticotropin-releasing hormone; *ENG*, endoglin; *STATA3*, signal transducer and activator of transcription 3; *TGFB3*, transforming growth factor beta 3; *PSG3*, pregnancy-specific beta-1-glycoprotein 3; *NRP1*, neuropilin-1; *LRP2*, LDL receptor-related protein 2; *LRRFIP1*, LRR binding FLII interacting protein 1; *AREG*, amphiregulin; *ANGPTL4*, angiopoietin-like 4; *BDNF*, brain-derived neurotrophic factor; *CDH1*, cadherin 1; *COL4A2*, collagen type IV alpha 2 chain; *CPXM*, carboxypeptidase X, M14 family member; *CAV1*, caveolin-1; *HIF1A*, hypoxia-inducible factor 1-alpha; *JAG1*, jagged canonical Notch ligand 1; *VEGFA*, vascular endothelial growth factor A; *PI3K*, phosphoinositide 3-kinase; *STAT3*, signal transducer and activator of transcription 3; *GSK3B*, glycogen synthase kinase 3 beta.

There were 11, 19, and 61 nodes identified from datasets I, II and III, respectively, and their networks are illustrated in **Figures 5A** and **4B, C**. Among them, 16 nodes with interactive degree ≥8 (**Supplementary Table S6**) were classified as key nodes: *VEGFA*, *STAT3*, *LEP*, *CDH1*, caveolin-1 (*CAV1*), CD44 antigen (*CD44*), *TGFBR1*, *FLT1*, *HIF1A*, *TGFBR2*, *WNT2*, endoglin (*ENG*), brain-derived neurotrophic factor (*BDNF*), *GSK3B*, jagged canonical Notch ligand 1 (*JAG1*), and *PRL*. All of these 16 key nodes are related to vasculogenesis and angiogenesis through their pro-angiogenic or anti-angiogenic functions.

Of note is that *VEGFA*, as the hub gene, exhibited the highest interaction degree of 28. Furthermore, within the molecular interaction network comprising these 16 key nodes, *VEGFA* also possessed the most interactions (15 links) (**Figure 5D**). *VEGFA* is a target mRNA of the DEmiR miR-199a-5p, which was significantly upregulated in the sFGR placentae in our previous study (20). We therefore speculate that the expression of *VEGFA* may also be altered accordingly.

*HIF1A* is the central upstream regulator of *VEGFA* and also serves as another target mRNA of miR-199a-5p, with six interactive links in the network. Previous studies demonstrated that *LEP*, *sFLT1*, *ENG*, *JAG1*, and *BDNF* are important downstream target genes of *HIF1A*, primarily involved in physiological processes such as angiogenesis and vascular regulation (46–50). In addition, *HIF1A* was inhibited by endoplasmic reticulum lectin (OS9) in a possible molecular pathway in our previous study in sFGR placentae (22).

Therefore, based on their central positions in the network, established regulatory relationships, and relevance to placental pathophysiology, *VEGFA* and *HIF1A* were prioritized as key targets for subsequent mechanistic studies.

### 3.4 Validation studies

Our findings of *VEGFA* and *HIF1A* were further validated using RT-qPCR and IHC in 25 pairs of monochorionic, diamniotic (MCDA) twins (seven control pairs, 12 uncomplicated sFGR, and six complicated sFGR pairs). Their demographic characteristics are shown in **Supplementary Table S7**. The birth weight *z* score of the smaller twins in the sFGR group was significantly lower than that in the control group (median = −1.8 *vs*. −0.8; *p* < 0.001). The birth weight discrepancy between the pairs in the sFGR group was also significantly larger than that in the control group (median = 21% *vs*. 9%; *p* = 0.041).

**Figure 6:**
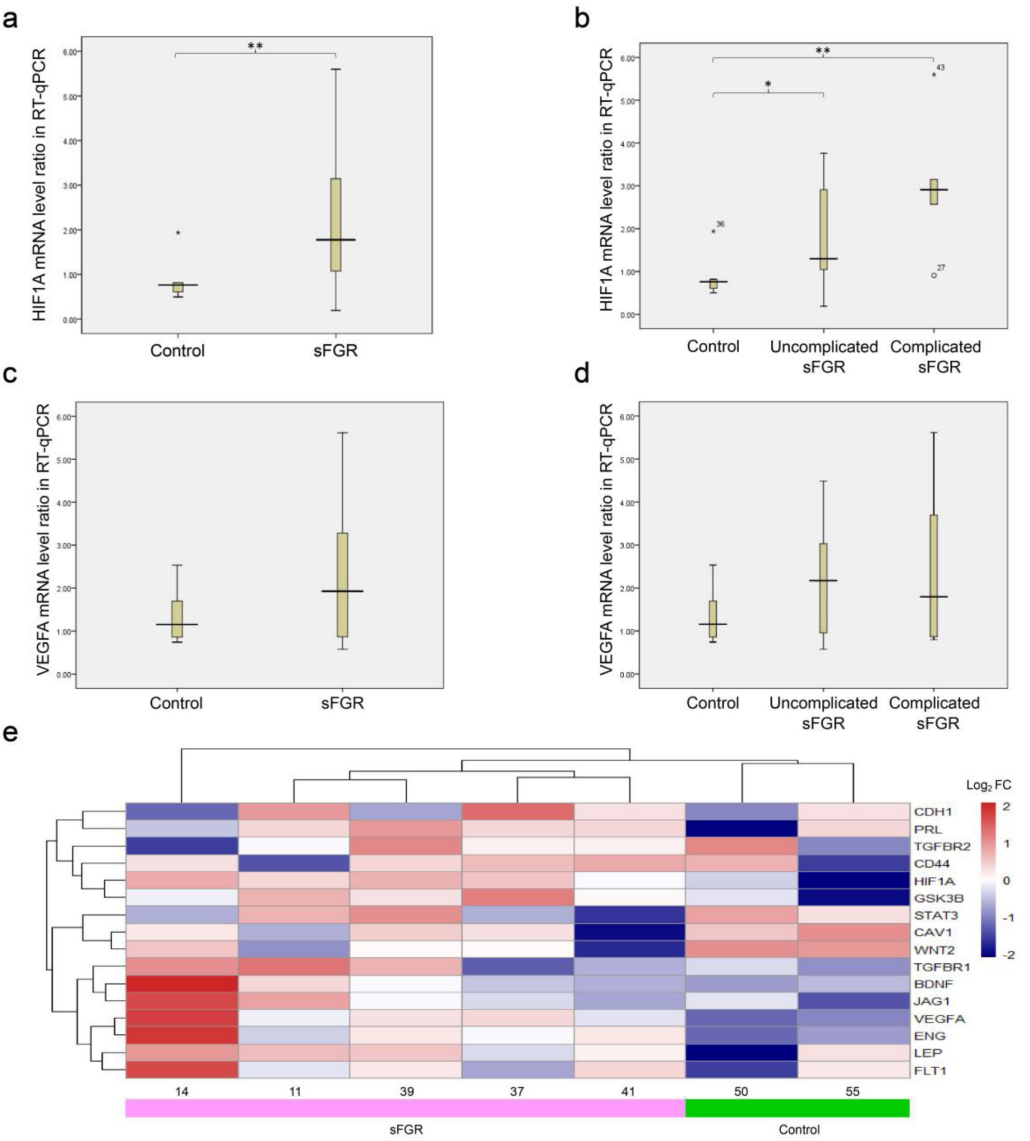
Transcriptional-level validation results. **(A, B)** Quantitative real-time PCR (RT-qPCR) ratio (smaller twin/larger co-twin) of *HIF1A*. **(C, D)** RT-qPCR ratio (smaller twin/larger co-twin) of *VEGFA*. **(E)** Heatmap of placental whole-genome RNA sequencing representing the log2FC of 16 key nodes between the selective fetal growth restriction (sFGR) group (cases 14, 11, 39, 37, and 41) and the control group (cases 50 and 55). *Asterisks* denote any pairwise comparisons that are significantly different. *p < 0.05; **p < 0.01. *HIF1A*, hypoxia-inducible factor 1-alpha; *VEGFA*, vascular endothelial growth factor A; *PRL*, prolactin; *CRH*, corticotropin-releasing hormone; *TGFBR2*, transforming growth factor beta receptor 2; *STATA3*, signal transducer and activator of transcription 3; *CAV1*, caveolin-1; *WNT2*, Wnt family member 2; *BDNF*, brain-derived neurotrophic factor; *JAG1*, jagged canonical Notch ligand 1; *LEP*, leptin; *FST1*, follistatin 1; *ENG*, endoglin.

The mRNA expression levels of *HIF1A* and *VEGFA* were analyzed using RT-qPCR. Compared with that in the control group, the *HIF1A* expression in sFGR twins was significantly upregulated (median = 1.78 *vs*. 0.76; *p* = 0.005) (**Figure 6A**). Among the subgroups, the expression level was highest in the complicated sFGR group, followed by the uncomplicated sFGR group (respective median values of 2.74 *vs*. 1.30 *vs*. 0.76; adjusted *p* < 0.01 for complicated sFGR and adjusted *p* < 0.05 for uncomplicated sFGR) (**Figure 6B**). In contrast, the expression of *VEGFA* in the sFGR group and its subgroups showed no significant difference compared with the control group (**Figures 6C, D**). These results between the sFGR groups and the control group were further compared with the results in five pairs of sFGR and two pairs of control twins using our own RNAseq dataset (22). Similarly, *HIF1A* and *VEGFA* were both upregulated in the RNAseq study, but only *HIF1A* had a significant result (*p* < 0.05) (**Figure 6E**).

**Figure 7:**
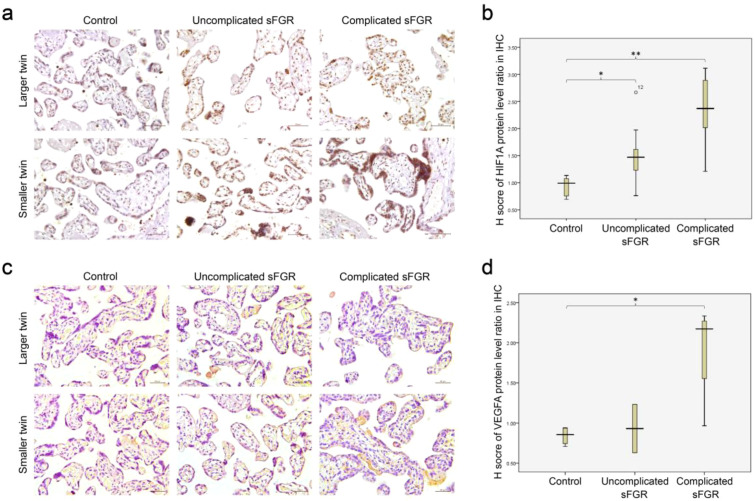
Protein level validation results. **(A)** Representative picture of the immunohistochemical (IHC) staining (×200) of *HIF1A* among different groups. **(B)** Quantitative IHC results of *HIF1A* among different groups. **(C)** Representative picture of the IHC staining (×200) of *VEGFA* among different groups. **(D)** Quantitative IHC results of *VEGFA* among different groups. In the box plot of *H*-score, the fold change of the protein expression level is presented as a ratio (smaller twin/larger twin) for comparison. *Asterisks* denote any pairwise comparisons that are significantly different. *p < 0.05; **p < 0.01. *sFGR*, selective fetal growth restriction; *HIF1A*, hypoxia-inducible factor 1-alpha; *VEGFA*, vascular endothelial growth factor A.

The protein expression levels of *HIF1A* and *VEGFA* were analyzed using IHC. A significant increase in the protein expression of *HIF1A* was observed in the placentae of sFGR twins, with the highest expression in the complicated sFGR group, followed by the uncomplicated sFGR group (respective median values of 2.37 *vs*. 1.47 *vs*. 0.99; adjusted *p* < 0.01 for complicated sFGR and adjusted *p* < 0.05 for uncomplicated sFGR) (**Figures 7A, B**). The protein expression of *VEGFA* was only statistically significantly upregulated in the complicated sFGR subgroup (median = 2.17 *vs*. 0.85; adjusted *p* = 0.021), but not in the uncomplicated sFGR subgroup (median = 0.93 *vs*. 0.85; *p* = 1.0) (**Figures 7C, D**).

## 4 Discussion

This study showed that hypoxia, vasculogenesis, and angiogenesis are the most significant pathophysiological pathways, whereas *HIF1A* and *VEGFA* are the key nodes of the molecular network in FGR. Upregulation of the transcriptional regulator *HIF1A* and the angiogenic factor *VEGFA* was validated using our sFGR model. The degree of upregulation also correlated with the clinical severity.

Angiogenesis and vasculogenesis play an important role in the development of the villous vasculature and the formation of terminal villi during development of the human placenta. Placental vascular growth begins early in pregnancy and continues throughout gestation (51, 52). The villous vasculature increases in number from the 21st day of development until the end of the first trimester. From the 26th week of gestation until term, villous vascular growth changes from branching to non-branching angiogenesis due to the formation of mature intermediate villi that specialize in gas and nutrient exchange. Reduced vascular development in the placental villi is a common event in FGR and sFGR pregnancies (53, 54).

*HIF1A* is a transcription factor that plays an important role in placental development. The placental expression levels of *HIF1A* were not consistent in previous studies. *HIF1A* expression was not altered or upregulated in FGR-affected pregnancies (55–57). In our validation study, both the *HIF1A* mRNA and protein expression were significantly increased in the placentae of sFGR pregnancies. *HIF1A* is oxygen-sensitive, which is induced by hypoxia and is rapidly degraded and inactivated during normoxia. In the FGR process, placental angiogenesis and vasculogenesis are poorer than that in normal pregnancy, making the placenta underperfused and hypoxic, which would stimulate *HIF1A* expression.

*VEGFA* is a downstream gene of *HIF1A*. *VEGFA* activates phospholipase C-γ, phosphoinositide 3-kinase, protein kinase B, Ras, proto-oncogene tyrosine-protein kinase Src, and mitogen-activated protein kinase to regulate cell proliferation, migration, survival, and vascular permeability (58). The expression of *VEGFA* in FGR studies is controversial, which has been reported as either downregulated or upregulated (59–61). In the early stage of placental vasculogenesis and angiogenesis, there is an increase in the expression of *VEGFA*. However, as pregnancy progresses, the expression of *VEGFA* decreases in the later non-branching angiogenesis. The increase of *VEGFA* expression in FGR and sFGR may be due to the stimulation of *HIF1A*. Taken together, the upregulation of *VEGFA* and *HIF1A* expression may be a compensatory response aimed at catching up with the placental development in sFGR pregnancies through enhancing angiogenesis (62).

In addition to the angiogenesis pathway, some other significant pathways were enriched in other studies, such as cell adhesion (22, 45), lysine degradation, and the HIF1 signaling pathway (24, 38). However, these pathways were not enriched in our study. This difference could be due to the small number of highly differentiated expressed gene inputs for our enrichment analysis. With our approach, although the number of inputs for enrichment will be reduced, the reliability can be increased significantly. There are a total of 76 genes participating in the HIF1 pathway in the KEGG database; however, many of the target genes of *HIF1A* were not defined as differentially expressed transcripts of FGR and sFGR for KEGG enrichment. Therefore, HIF1 signaling was not directly enriched in our results.

In our GO analysis, the CC extracellular space was enriched in all three datasets, indicating that the gene products from the placentae of FGR could potentially be detected in maternal blood. In previous studies, a number of placenta-derived serum markers, such as pregnancy-associated plasma protein A (PAPPA) (63, 64), A-disintegrin and metalloprotease 12 (ADAM12) (65, 66), and placental protein 13 (PP13), were measured in maternal blood (67, 68). However, these markers were not sensitive and specific enough for clinical prediction of FGR or gestational age (SGA) in singleton pregnancies. Potentially, our study may discover new placental markers for noninvasive detection to identify high-risk pregnancies before clinical presentation and even therapeutic targets of FGR to improve fetal morbidities or mortality.

The proteins encoded by our seven key nodes—*VEGFA*, *LEP*, *CD44*, *FLT1*, *BDNF*, *ENG*, and *PRL*—have been well documented to be secreted into the blood circulation, supporting their potential as noninvasive biomarkers for sFGR, which merits validation in large-scale cohorts. Notably, an integrative bioinformatics analysis of PE by Liu et al. aligns with and extends our findings (26). The study demonstrated that *LEP* mRNA is significantly upregulated in PE placentae and serves as a potential diagnostic biomarker for this condition, which echoes our identification of *LEP* as a core key node in sFGR. This convergence confirms the shared pathological mechanisms underlying placental dysfunction in both disorders, particularly in pathways governing angiogenesis and hypoxia response. Conversely, the same PE study identified *CRH* as one of the top 10 upregulated genes, a finding that contrasts with our transcriptomic and validation data: we observed no significant differential expression of *CRH* in FGR/sFGR placental tissues. This discrepancy highlights that, while FGR and PE overlap in core pathophysiological pathways, they exhibit distinct molecular regulatory signatures, underscoring the value of multi-molecular combinatorial panels for the clinical differentiation of these two frequently comorbid placental-related complications. Furthermore, our results revealed that the magnitude of *HIF1A* and *VEGFA* upregulation was greater in the complicated sFGR subgroup than in the uncomplicated sFGR subgroup, indicating more severe placental hypoxia and impaired angiogenesis/vasculogenesis in complicated sFGR. These observations also imply that secretory angiogenic markers may correlate with the sFGR subtype, which represents an important direction for future research on early pregnancy classification of sFGR types. In addition, in our results, the degree of *HIF1A* and *VEGFA* upregulation was higher in the complicated sFGR subgroup compared with the uncomplicated sFGR subgroup, indicating that hypoxia, poorly developed angiogenesis, and vasculogenesis are more severe in the placentae of complicated sFGR compared with uncomplicated sFGR. These results also imply that secretory angiogenic markers may be related to the type of sFGR. Whether these can be used to classify the type of sFGR in early pregnancy needs to be further studied.

This is the first comprehensive study analyzing dysregulated placental genes from non-target whole-genome transcriptome profiling of placentae in FGR and sFGR pregnancies. We acknowledge the central role of the hypoxia–angiogenesis axis in the pathophysiology of sFGR and validated these findings in our own cohort. Importantly, our study also provided novel insights. Firstly, using the etiologically well-controlled MC sFGR model, we demonstrated that the degree of activation of the *HIF1A*–*VEGFA* hub was directly associated with the clinical indicators of fetal hemodynamic deterioration, providing new evidence to support these molecules as potential quantitative markers for disease severity. Secondly, our network analysis highlighted several key secreted nodes, proposing novel candidate combinations for the development of noninvasive, peripheral blood-based diagnostic panels using a multi-marker approach. The integrated transcriptomic datasets used in our study were derived from multiple independent studies with heterogeneous designs, posing an inherent challenge for integrative analyses. Potential sources of heterogeneity include differences in the study populations, gestational age at delivery, and placental sampling sites. Such variability may introduce confounding effects and influence the magnitude of observed gene expression changes. To mitigate these effects, we applied strict inclusion criteria and focused on well-defined sFGR phenotypes. Importantly, our integrative strategy emphasized consistently dysregulated genes and pathways that were reproducibly identified across datasets, thereby enhancing the robustness of the core findings.

However, our current study also has several limitations. Firstly, a major limitation is that the placental transcriptomic and molecular analyses were based on tissues collected at delivery, reflecting the terminal state of the placenta after prolonged adaptation and compensation rather than the dynamic regulation of early placental angiogenesis and trophoblast invasion. Key molecules such as *HIF1A* and *VEGFA* exhibit marked gestational age-dependent expression with distinct roles in early and late placental development. Consequently, the dysregulated molecular networks identified are more likely to represent compensatory responses of the late placenta to chronic hypoxia than early developmental abnormalities preceding clinical manifestation. Longitudinal studies or placental sampling in early pregnancy are therefore required to determine whether similar disturbances occur during critical windows of placental formation. Secondly, the placental sampling location is an important determinant of molecular findings, particularly in sFGR placentae, where a marked heterogeneity in perfusion and villous development exists across different regions (69). In this study, a standardized protocol targeted well-vascularized regions while avoiding infarcted or calcified areas to reduce tissue heterogeneity and focus on hypoxic–angiogenic signaling within viable tissues. However, this approach may not have fully captured poorly perfused regions. Consequently, our findings mainly reflect molecular changes in hemodynamically stressed but viable placental areas rather than an average across the entire placenta. Thirdly, our study employed multiple strategies to control for confounding factors. Strict data screening and the use of MC twin characteristics effectively minimized potential confounders. In addition, unified data normalization and standardized cross-validation enhanced the robustness. Residual confounding effects cannot be completely excluded. Factors such as the delivery mode and the *in vitro* fertilization (IVF) status, which may influence placental gene expression, were not fully adjusted for. Future studies should adopt prospective matched cohort designs and expand sample sizes to enhance statistical power. Fourthly, both the literature-integrated and our own cohort included relatively small sample sizes, particularly for sFGR cases, which may have limited the statistical power and the generalizability of the findings. Finally, the heterogeneity of the study population may render our conclusions inapplicable to other types of pregnancies or FGR subtypes with different etiologies. Moreover, in singleton FGR pregnant studies, although the studied tissues were only placentae, intrinsic fetal genetic factors and maternal environmental factors may also contribute to transcriptomic changes of placentae. Hence, the differential placental gene expression between FGR cases and controls reflects not only the dysfunctional status of the placentae of FGR cases but also the dysregulated fetal and maternal factors of FGR.

## 5 Conclusion

In conclusion, this study has demonstrated that hypoxia-, vasculogenesis-, and angiogenesis-associated pathways via the molecular networks of *HIF1A* and *VEGFA* play an important role in the placental pathomechanism of FGR.

## Data availability statement

The original contributions presented in the study are included in the article/**Supplementary Materials**. Further inquiries can be directed to the corresponding author.
