# Supplementary material for: Common and recurrent dysregulated molecular network of placental hypoxia and associated vasculogenesis and angiogenesis in fetal growth restriction
Source: Front Endocrinol (Lausanne). 2026 Feb 25;17:1729898. doi: 10.3389/fendo.2026.1729898 (PMC12975423; doi:10.3389/fendo.2026.1729898)
Supplement: Supplementary file 1 [file Table1.docx]

**Supplementary Table S1 Non-target RNA detection in singleton pregnancies complicated by [fetal growth restriction, and in twin pregnancies complicated by selective fetal growth restriction](https://obgyn.onlinelibrary.wiley.com/doi/pdf/10.1002/uog.20093)**

| Publication | Country | Study  type^a^ | Method:  case number^b^ | Recruited patients | Control cases | Excluded  cases^c^ | Cut-off values | Total DE genes | Significant genes^d^ | | Associated pathway | RT-qPCR validation:  case number^b^ and results |
| --- | --- | --- | --- | --- | --- | --- | --- | --- | --- | --- | --- | --- |
|  |  |  |  | Diagnostic criteria | Matched by GA |  |  |  | ↑ | ↓ |  |  |
| **Singleton studies** | | | | | | | | | | | | |
| Diplas AI  2009^1^ | USA | RNA & epigenomics | qPCR assays:  7/10 | EFW<3^rd^  abnormal UA^e^ | NA | Congenital FGR | p<0.05 | 5↑  4↓ | *PHLDA2^M^, ILK2, NNAT^P^, CCDC86, PEG10^P^* | *PLAGL1^P^, DHCR24, ZNF331^P^, CDKAL1* | NA | NA |
| Struwe E  2010^2^ | Germany | mRNA | Microarray:  10/10 | BW <10^th^  at least one parameter of pathological ultrasound 1^f^ | Yes | PE, CA, IUIF | p<0.05 | 132 ↑  25↓ | *LEP, IGFBP1, CRH, PRL* |  | NA | 33/35,  Positive |
| Sabri A  2014^3^ | Australia | mRNA | Microarray: 4/5 | BW <10^th^ | Yes | NA | FC>2 p<0.05 | 199↑  139↓ | *CPXM2, RAB3B, GNGT1* | *TXNDC5, AREG, LRRFIP1, LRP2* | dorsoventral axis formation, adherens junction | 4/5,  Positive |
| Gremlich S  2014^4^ | Switzerland | RNA | PCR-Select cDNA subtraction: 5/5 | EFW<10^th^ + BW <10^th^ | No | PE, Dia, FM, IUIF | p<0.05 | 9 | *NEAT1* | *PSG3* | NA | 12/12,  Positive |
| Borg AJ  2015^5^ | Australia | mRNA | PCR array:  26/27 | BW<10^th^  at least two parameters of pathological ultrasound 2^g^ | Yes | PA, ROM, PE, AD, FM, IUIF | p<0.05 | 3 ↓ |  | *STAT3* | JAK–STAT signaling pathway | 26/27,  Positive |
| Madeleneau D  2015^6^ | France | mRNA | Microarray:  12/12 | BW <10^th^  pathological ultrasound 3^h^ | NA | PE, Dia, AD, CA, FM | FC>2 p<0.05 | 206 ↑  430 ↓ | *LEP, IGFBP1, RBP4* |  | lipid metabolism | 26/21,  Positive |
| Maulik D 2016^7^ | USA | mRNA | Microarray:  7/7 | EFW <10^th^  absent end-diastolic flow in UA | Yes | PA, ROM, PE, Dia, CA, FM | FC>2 p<0.05 | 1↓ |  | *NRP1* |  | 7/7,  Positive |
| Nguyen TP  2018^8^ | Australia | mRNA | PCR array:  25/25 | BW<10^th^  at least two parameters of pathological ultrasound 2^g^ | Yes | PA, ROM, PE, AD, FM, IUIF | p<0.05 | 1↑  5↓ |  | *TGFB3* |  | 25/25,  Positive |
| Paauw ND  2018^9^ | Netherlands | RNA & epigenomics | ChIP-seq: 5/4  RNA-seq: 5/4 | EFW <3^rd^  UA-PI >95^th^ centile | No | NA | p<0.05 | 569↑  521↓ | *MIR210HG, ZNF114, SH3BP5, LEP, HTRA4, AMD1, FLT1, SLC2A1, QPCT, RASAL1, MME, HK2, C1orf21, VWCE, SLC9A3R2, SERPIND1, ENG, NIM1K, TPBG, MGAT3, CDK19, NTF4, ERRFI1, MAVS, DNAJC1, BIN2, DPP7, GBA, ARMS2, FOS, HN1L, SASH1, PIP5KL1, ST8SIA6* | *CSH2, FAM26D, GH2, CSH1, CSHL1，OR7E14P, S100A9, CLDN1, FAM26E, MAD2L2, GSTK1, RCAN1, FRZB, EGFEM1P, RNF29, MIR762HG, ACOXL, ARHGAP42, ST20, COL9A1, ARL4A, SYNPO2L, TMEM139, S100A8, GPR155, TLDC1* | HIF-1-alpha transcription factor network | NA |
| Ranzil S  2019^10^ | Australia | mRNA | fluidigm dynamic array:  23/42 | BW <10^th^  any one parameter of pathological ultrasound 2^g^ | Yes | PA, ROM, PE, Dia, AD, CA, FM, IUIF | p<0.05 | 2↓ ,  3↑ |  | *TPH2, SLC6A4* | 5- hydroxytryptamine synthetic pathway | NA |
| Chabrun F 2020^11^ | France | RNA & epigenomics | DNA methylation microarray:  28/8  RNA microarray: 28/8 | BW <10^th^  pathological ultrasound 3^h^ | No | NA | p<0.05 | 579 | *PAPPA2, BCL6, SLC2A1, AFAP1, ALDOA, ALKBH5, CALM1, DGKZ, DLX5, FLNB, LIMCH1, PEA15, PLEKHA2, RRAD* | *AP2A1, UNKL, WSB1, C1QTNF1, FOXK1, PDP2, PDXK, RALGPS1, SFRS8, UCKL1, USP5* | NAD-binding, histone acetylation, mTOR signaling pathway | NA |
| **Twin studies** | | | | | | | | | | | | |
| Schrey S  2013^12^ | USA | RNA & epigenomics | PCR array:  10/5 | MC  BW difference>=20% | NA | placental tumors, PE, Dia, FM, IUIF | p<0.05 | 3↑ | *FLT1, ENG, LEP* | NA | NA | 7/0,  Positive |
| He Z  2016^13^ | China | RNA & epigenomics | LHC-BS:  7/6  Mass spectrometry:  7/? | MC  one twin’s BW< 10^th^ percentile | NA | maternal complication, TTTs, FD, | p<0.05 | NA | NA | NA | DNA binding activity, organismal development process | 11/?,  Negative |
| Wen H  2017^14^ | China | ncRNA | miRNA microarray:  2/1 | MC  EFW discordance > 20%^i^ | NA | maternal complication, TTTs, FM, | FC>2 p<0.05 | 14 | *miR-373‑3p, miR‑338‑5p, miR‑590‑5p* | *miR-1,*  *miR‑370‑3p, miR‑5189‑5p* | organ size, cell differentiation, cell proliferation and migration | 15/15,  Positive |
| Li L  2019^15^ | China | ncRNA | miRNA seq: 6/6 | MC  EFW of one foetus < 10^th^ percentile+ EFW discordance > 25%^i^ | No | PE, Dia, TTTs, FD, CA, FM | p<0.05 | 11 miR↑  7 miR↓ | *miR‐210‐3p* | NA | angiogenesis, fibroblast growth factor stimulation, cell adhesion and migration | 18/18,  Positive |
| Meng Meng  2020^16^ | Hong Kong | ncRNA | miRNA microarray: 5/3 | MC  EFW of one foetus < 10th percentile +BW of one foetus < 10^th^ percentile | Yes | FD, Misc, CA, BW of both foetuses <10^th^ percentile | FC>1.5 p<0.05 | NA | *miR-199a-5p* | NA | angiogenesis, oxidative stress, mitochondrial damage and dysfunction | 13/8,  Positive |
| Wei Li  2020^17^ | Hong Kong | mRNA | RNA seq:  5/2 | MCDA  EFW of one fetus <3^rd^ centile, or when there were at least two out of four contributory parameters (EFW or AC of one foetus <10^th^ centile, EFW discordance >=25^i^, UA PI of the smaller twin >95^th^ centile)+ double confirmed with BW | Yes | FD, Misc, CA, BW of both fetuses <10^th^ percentile | p<0.05 | 610 ↑ 819 ↓ | *OS9, M6PR* | NA | phagosome maturation, unfolded protein response | 13/6,  Positive |
| Yi Zhang  2020^18^ | China | RNA & epigenomics | DNA immunoprecipitation-chip: 4/4  RNA seq: 4/4 | MCDA  BW of one fetus <10^th^ percentile, and intertwin discordance > 25% | No | maternal complication, TTTs, FD, FM | p<0.05 | 181 ↑  614 ↓ | NA | *ANGPTL4* | HIF-1 signaling  pathway | 13/18,  Positive |

DE, differential expressed; FC, fold change; GA, gestational age; BW, birthweight; EFW, estimated fetal weight; UA, umbilical artery; UtA, uterine artery; PI, pulsatility index; abdominal circumference, AC; MC, monochorionic; MCDA, monochorionic diamnionic; LHC-BS, liquid hybridization capture-based bisulfite sequencing; NA, not applicable.

^a^Abbreviation of study type: RNA & epigenomics, RNA study overlapped with epigenomics; mRNA, mRNA only study; RNA, mRNA and non-coding RNA study; ncRNA, non-coding RNA only study.

^b^Case number: FGR or sFGR / control

^c^Abbreviation of disease: PA, placental abruption; ROM, rupture of the fetal membranes; PE, preeclampsia; Dia, diabetes; AD, addictions (such as tobacco or drug usage); TTTs, twin‑to‑twin transfusion syndrome; FD, foetal death; Misc, miscarriage; CA, chromosomal abnormalities; FM, fetal malformation; IUIF, intrauterine infection.

^d^Imprinted genes were shown with superscript M or P (M: maternally expressed, P: paternally expressed).

^e^Abnormal UA: absent or reversed end-diastolic flow in the umbilical artery

^f^Pathological ultrasound 1: pulsatility index in one uterine artery >1.2 or early diastolic notches.

^g^Pathological ultrasound 2: abnormal umbilical artery Doppler flow velocimetry; oligohydramnios; and fetal growth asymmetry.

^h^Pathological ultrasound 3: a notch observed by Echo-Doppler in at least one uterine artery and with Doppler abnormalities on umbilical Doppler and/or cerebral Doppler and/or ductus venosus.

^i^EFW discordance=[(EFW of larger twin‑ EFW of smaller twin)/ EFW of larger twin]

**References**

1. Diplas AI, Lambertini L, Lee MJ, Sperling R, Lee YL, Wetmur J, Chen J. Differential expression of imprinted genes in normal and IUGR human placentas. *Epigenetics* 2009;**4**:235-240.

2. Struwe E, Berzl G, Schild R, Blessing H, Drexel L, Hauck B, Tzschoppe A, Weidinger M, Sachs M, Scheler C, Schleussner E, Dotsch J. Microarray analysis of placental tissue in intrauterine growth restriction. *Clinical endocrinology* 2010;**72**:241-247.

3. Sabri A, Lai D, D'Silva A, Seeho S, Kaur J, Ng C, Hyett J. Differential placental gene expression in term pregnancies affected by fetal growth restriction and macrosomia. *Fetal Diagn Ther* 2014;**36**:173-180.

4. Gremlich S, Damnon F, Reymondin D, Braissant O, Schittny JC, Baud D, Gerber S, Roth-Kleiner M. The long non-coding RNA NEAT1 is increased in IUGR placentas, leading to potential new hypotheses of IUGR origin/development. *Placenta* 2014;**35**:44-49.

5. Borg AJ, Yong HE, Lappas M, Degrelle SA, Keogh RJ, Da Silva-Costa F, Fournier T, Abumaree M, Keelan JA, Kalionis B, Murthi P. Decreased STAT3 in human idiopathic fetal growth restriction contributes to trophoblast dysfunction. *Reproduction* 2015;**149**:523-532.

6. Madeleneau D, Buffat C, Mondon F, Grimault H, Rigourd V, Tsatsaris V, Letourneur F, Vaiman D, Barbaux S, Gascoin G. Transcriptomic analysis of human placenta in intrauterine growth restriction. *Pediatr Res* 2015;**77**:799-807.

7. Maulik D, De A, Ragolia L, Evans J, Grigoryev D, Lankachandra K, Mundy D, Muscat J, Gerkovich MM, Ye SQ. Down-regulation of placental neuropilin-1 in fetal growth restriction. *Am J Obstet Gynecol* 2016;**214**:279 e271-279 e279.

8. Nguyen TPH, Yong HEJ, Chollangi T, Brennecke SP, Fisher SJ, Wallace EM, Ebeling PR, Murthi P. Altered downstream target gene expression of the placental Vitamin D receptor in human idiopathic fetal growth restriction. *Cell Cycle* 2018;**17**:182-190.

9. Paauw ND, Lely AT, Joles JA, Franx A, Nikkels PG, Mokry M, van Rijn BB. H3K27 acetylation and gene expression analysis reveals differences in placental chromatin activity in fetal growth restriction. *Clin Epigenetics* 2018;**10**:85.

10. Ranzil S, Ellery S, Walker DW, Vaillancourt C, Alfaidy N, Bonnin A, Borg A, Wallace EM, Ebeling PR, Erwich JJ, Murthi P. Disrupted placental serotonin synthetic pathway and increased placental serotonin: Potential implications in the pathogenesis of human fetal growth restriction. *Placenta* 2019;**84**:74-83.

11. Chabrun F, Huetz N, Dieu X, Rousseau G, Bouzille G, Chao de la Barca JM, Procaccio V, Lenaers G, Blanchet O, Legendre G, Mirebeau-Prunier D, Cuggia M, Guardiola P, Reynier P, Gascoin G. Data-Mining Approach on Transcriptomics and Methylomics Placental Analysis Highlights Genes in Fetal Growth Restriction. *Front Genet* 2019;**10**:1292.

12. Schrey S, Kingdom J, Baczyk D, Fitzgerald B, Keating S, Ryan G, Drewlo S. Leptin is differentially expressed and epigenetically regulated across monochorionic twin placenta with discordant fetal growth. *Molecular Human Reproduction* 2013;**19**:764-772.

13. He Z, Lu H, Luo H, Gao F, Wang T, Gao Y, Fang Q, Wang J. The promoter methylomes of monochorionic twin placentas reveal intrauterine growth restriction-specific variations in the methylation patterns. *Scientific reports* 2016;**6**:20181.

14. Wen H, Chen L, He J, Lin J. MicroRNA expression profiles and networks in placentas complicated with selective intrauterine growth restriction. *Mol Med Rep* 2017;**16**:6650-6673.

15. Li L, Huang X, He Z, Xiong Y, Fang Q. miRNA-210-3p regulates trophoblast proliferation and invasiveness through fibroblast growth factor 1 in selective intrauterine growth restriction. *Journal of Cellular and Molecular Medicine* 2019;**23**:4422-4433.

16. Meng Meng, Yvonne Kwun Yue Cheng, Ling Wu, Piya Chaemsaithong, Maran Bo Wah Leung, Stephen Siu Chung Chim, Daljit Singh Sahota, Wei Li, Liona Chiu Yee Poon, Chi Chiu Wang, Leung TY. Whole genome miRNA profiling revealed miR-199a as potential placental pathogenesis of selective fetal growth restriction in monochorionic twin pregnancies. *Placenta* 2020;**92**:44-53.

17. Wei Li, Claire Yik Lok Chung, Chi Chiu Wang, Ting Fung Chan, Maran Bo Wah Leung, Oi Ka Chan, Ling Wu, Kubi Appiah, Piya Chaemsaithong, Yvonne Kwun Yue Cheng, Liona Chiu Yee Poon, Leung TY. Monochorionic twins with selective fetal growth restriction: insight from placental whole transcriptome analysis *Am J Obstet Gynecol* 2020.

18. Zhang Y, Zheng D, Fang Q, Zhong M. Aberrant hydroxymethylation of ANGPTL4 is associated with selective intrauterine growth restriction in monochorionic twin pregnancies. *Epigenetics* 2020:1-13.
