# Supplementary material for: Common and recurrent dysregulated molecular network of placental hypoxia and associated vasculogenesis and angiogenesis in fetal growth restriction
Source: Front Endocrinol (Lausanne). 2026 Feb 25;17:1729898. doi: 10.3389/fendo.2026.1729898 (PMC12975423; doi:10.3389/fendo.2026.1729898)
Supplement: Supplementary file 7 [file Table7.docx]

**Supplementary Table S7** **Demographic characteristics of 18 pairs of monochorionic diamniotic twins complicated with sFGR compared with seven pairs of normal monochorionic diamniotic twins in RT-qPCR and IHC staining**

| **Variables** | **Control (n=7)** | **sFGR (n=18)** | ***P*** |
| --- | --- | --- | --- |
| Maternal age (years) | 34.8 (30.9 ─ 35.9) | 31.7 (29.9 ─ 35.3) | 0.263 |
| Parity (n, (%)) |  |  |  |
| Nulliparous | 2 (28.6) | 15 (83.3) | 0.023 |
| Parous | 5 (71.4) | 3 (16.7) |  |
| Maternal body mass index (kg/m^2^) |  |  |  |
| at first trimester | 20.3 (19.2 ─ 25.4) | 21.8 (19.6 ─ 24.2) | 0.545 |
| at delivery | 27.5 (24.5 ─ 32.1) | 27.1 (24.7 ─ 31.9) | 0.832 |
| Gestational age at delivery (weeks) | 35.0 (33.9 ─ 36.4) | 35.9 (33.7 ─ 36.5) | 0.628 |
| Mode of delivery (n, (%)) |  |  |  |
| normal vaginal delivery | 0.0 (0.0) | 1.0 (5.6) | 0.687 |
| elective caesarean section | 2.0 (28.6) | 7.0 (38.9) |  |
| emergency caesarean section | 5.0 (71.4) | 10.0 (55.6) |  |
| Subtype of sFGR |  |  |  |
| uncomplicated sFGR | NA | 12 (66.7) | NA |
| complicated sFGR | NA | 6 (33.3) |  |
| Estimated fetal weight (g) |  |  |  |
| smaller twin | 1387(1212 ─ 1652) | 1079 (1664 ─ 1850) | 0.423 |
| larger twin | 1482 (1341 ─ 2142) | 2084 (1553 ─ 2299) | 0.389 |
| estimated fetal weight discrepancy (%)^a^ | 19 (9 ─ 23) | 18 (13 ─ 32) | 0.458 |
| Birth weight (g) |  |  |  |
| smaller twin | 2060.0 (1745.0 ─ 2345.0) | 1945.0 (1157.0 ─ 2065.0) | 0.238 |
| larger twin | 2060.0 (1970.0 ─ 2420.0) | 2267.5 (1827.8 ─ 2480.0) | 0.699 |
| birthweight discrepancy (%)a | 9 (7 ─ 13) | 21 (11 ─ 30) | 0.041 |
| Birthweight Z score |  |  |  |
| smaller twin | -0.8 (-1.0 to -0.8) | -1.8 (-1.9 to -1.6) | <0.001 |
| larger twin | -0.4 (-0.6 to 0.0) | -0.7 (-1.0 to -0.4) | 0.220 |
| Fetal sex |  |  |  |
| Male | 3.0 (42.9) | 8.0 (44.4) | 1.000 |
| Female | 4.0 (57.1) | 10.0 (55.6) |  |
| Cord arterial pH <7.2 |  |  |  |
| smaller twin | 0.0 (0.0) | 2.0 (11.1) | 1.000 |
| larger twin | 0.0 (0.0) | 0.0 (0.0) | NA |
| 1 minute Apgar score <7 |  |  |  |
| smaller twin | 2.0 (28.6) | 3.0 (16.7) | 0.597 |
| larger twin | 1.0 (14.3) | 1.0 (5.6) | 0.490 |
| 5 minute Apgar score <7 |  |  |  |
| smaller twin | 0.0 (0.0) | 0.0 (0.0) | NA |
| larger twin | 0.0 (0.0) | 0.0 (0.0) | NA |
| Admission to NICU |  |  |  |
| smaller twin | 5.0 (71.4) | 13.0 (72.2) | 1.000 |
| larger twin | 3.0 (42.9) | 11.0 (61.1) | 0.656 |

All continuous data were expressed as median and interquartile range (25^th^ percentile ─ 75^th^ percentile) or number (percentage within each type). Chi-square test was used for comparison of categorical variables. Mann-Whitney U test was used to compare the continuous variables.

^a^estimated fetal weight or birth weight discrepancy (%)=(estimated fetal weight or birth weight of larger twin - estimated fetal weight or birth weight of smaller twin) ×100 / estimated fetal weight or birth weight of larger twin

*RT-qPCR*, quantitative real-time polymerase chain reaction; *IHC*, immunohistochemistry; *NA*, not applicable; *NICU*, neonatal intensive care unit; *sFGR*: selective fetal growth restriction.
